# Correlation and consistency between two detection methods for serum 25 hydroxyvitamin D levels in human venous blood and capillary blood

**DOI:** 10.3389/fnut.2024.1291799

**Published:** 2024-06-11

**Authors:** Yutong Xing, Kaixi Wang, Xinyu Ma, Huifeng Zhang, Xiaoyu Tian

**Affiliations:** Department of Paediatrics, The Second Hospital Affiliated to Hebei Medical University, Shijiazhuang, China

**Keywords:** 25 hydroxyvitamin D, venous blood, capillary blood, detection methods, consistency evaluation

## Abstract

**Introduction:**

The study assessed the correlation and concordance of 25-hydroxyvitamin D [25(OH)D] levels in capillary and venous plasma collected simultaneously after vitamin D3 supplementation in 42 healthy adults. They were randomly divided into three groups by random number table method. Group A took 1,000 IU vitamin D3 daily, group B took 10,000 IU vitamin D3 every 10 days, and group C took 30,000 IU vitamin D3 every 30 days until the end of the 12th month. Venous blood serum 25(OH)D level was detected by chemiluminescence immunoassay (CLIA) and mass spectrometry (LC-MS) at day 1, day 14, day 28, month 6, and month 12 respectively, the capillary blood serum 25(OH)D level was detected by chemiluminescence immunoassay (CLIA) at the same time. Pearson correlation analysis and linear regression analysis were employed to investigate the relationship and transformation equation between the findings of the two samples and the results obtained from different detection methods within the same sample. The Bland-Altman method, Kappa analysis, and receiver operating characteristic (ROC) curve were utilized for assessing consistency, sensitivity, and specificity.

**Results:**

The three groups all reached a stable peak at 6 months, and the average levels of the three groups were 49.21, 42.50 and 43.025 nmol/L, respectively. The average levels of group A were higher than those of group B and group C (*P* < 0.001). The mean values of serum 25(OH)D measured by LC-MS and CLIA in 42 healthy adults were 45.32 nmol/L and 49.88 nmol/L, respectively, and the mean values of 25(OH)D measured by LC-MS in capillary blood were 52.03 nmol/L, and the difference was statistically significant (*P* < 0.001). Pearson correlation analysis showed that the linear fitting formula of scatter data was as follows: venous 25(OH)D concentration (nmol/L) = 1.105 ^*^ capillary 25(OH)D concentration −7.532 nmol/L, R^2^ = 0.625. Good agreement was observed between venous and corrected capillary 25(OH)D levels in clinical diagnosis (Kappa value 0.75). The adjusted serum 25(OH)D in capillary blood had a high clinical predictive value.

**Conclusions:**

The agreement between the two methods is good when the measured 25(OH)D level is higher. Standardized capillary blood chemiluminescence method can be used for 25(OH)D detection.

## 1 Introduction

Vitamin D exists in many forms in the body's circulation and participates in the body's metabolism. The two important forms of vitamin D are 25(OH)D2(Ergocalciferol) and 25(OH)D3(Hydroxycholecalciferol). The body cannot synthesize 25(OH)D2 and can only obtain it through food or supplements. Therefore, most of the 25(OH)D detected in serum is 25(OH)D3, and only a small number of people whose serum contains 25(OH)D2 can reach detectable levels (1, 2).

1, 25-dihydroxyvitamin D [1,25 (OH) 2D] is the active form of vitamin D in the body, but its content in the human body is very small and difficult to detect. Circulating 25-hydroxyvitamin D [25 (OH)D, including 25(OH)D2 and 25(OH)D3] is one of the main metabolic forms of vitamin D in the human body (3). The stable form and long half-life are the best indicators to evaluate the nutritional status of vitamin D in the human body (4). At present, the detection methods of serum 25(OH)D include liquid chromatography-tandem mass spectrometry, LC-MS/MS), electrochemiluminescence immunoassay (ECLISA), etc. LC-MS/MS can simultaneously detect multiple vitamin D variants such as 25(OH)D2 and 25(OH)D3, with strong specificity and high sensitivity. It is currently recognized as the gold standard method for 25(OH)D detection, and the measurement results are accurate and reliable. However, the requirements for laboratory equipment, personnel and measurement support conditions are high, and the detection technology is more complex and relatively expensive. Benefits of ECLIA include high throughput, labor savings, and real-time reporting of results. At present, the ECLIA method is widely used in medical institutions and is the only method for the detection of 25(OH)D in capillary serum analyzers. The specificity and detection principle of the two methods for detecting venous serum 25(OH)D mentioned in this paper are different.

**Figure 1:**
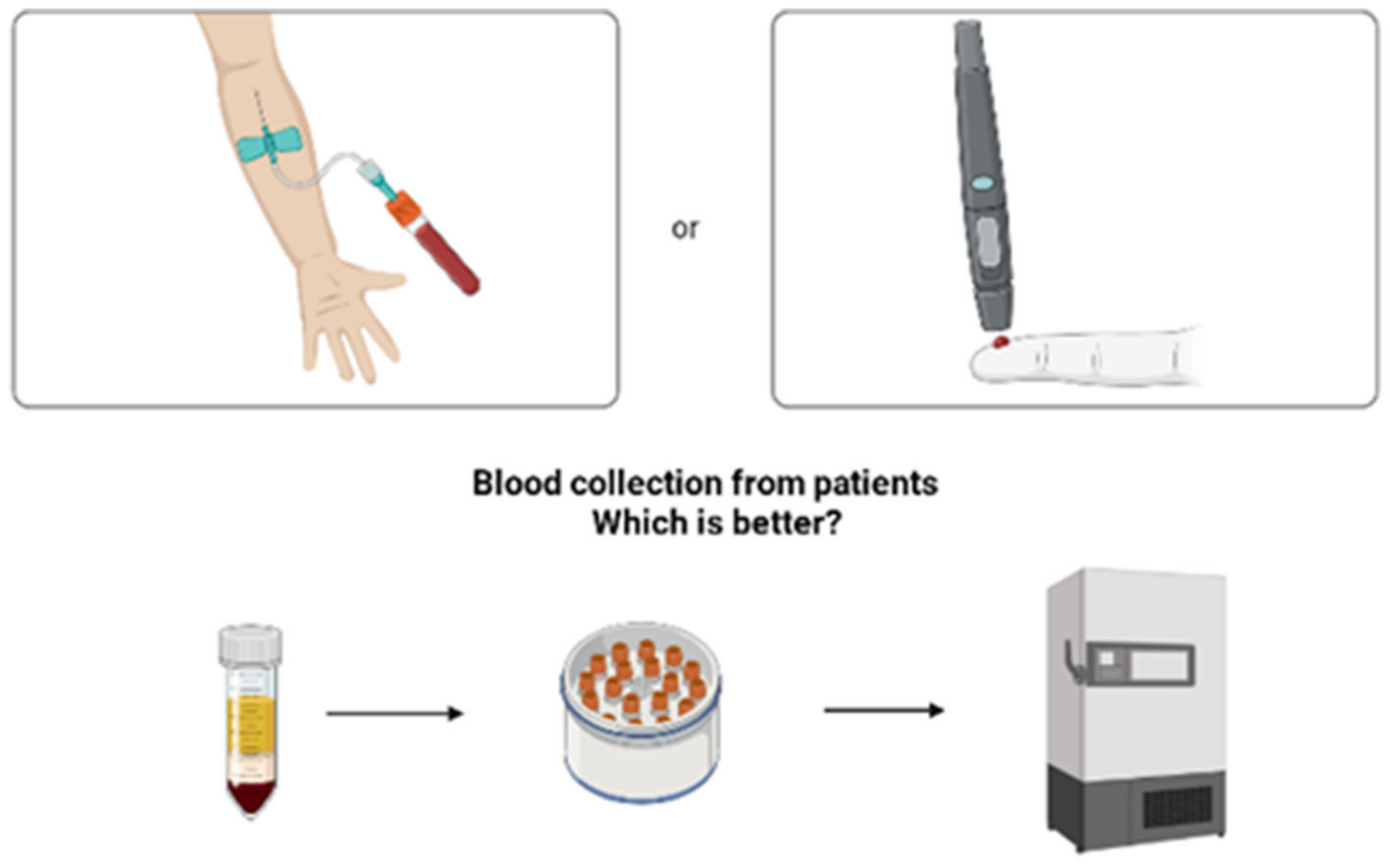
Deciding the better alternative for blood collection.

To determine 25(OH)D levels in body fluids, serum samples must be collected for analysis. For sample collection, different blood collection methods can be used clinically to obtain two blood samples: venous blood and capillary blood. Venous blood is widely used in clinical practice and is a long-acting blood collection method. However, for patients who are not suitable or unable to perform intravenous blood collection, such as children, extremely obese people, patients with severe burns, and patients with advanced cancer, it is more convenient to use the capillary tips of the heels and fingertips for blood collection. Different collection methods may affect the interpretation of the final data (Figure 1).

Relevant studies have shown that there is a good correlation between the levels of metabolites in venous blood and fingertip blood. However, the specific values that need to be converted may differ (5). It is important to explore effective and convenient methods to determine vitamin D status in order to timely detect vitamin D deficiency and supplement vitamin D. Therefore, our study aimed to analyze the effectiveness of two blood collection methods for 25(OH) D detection in healthy people during vitamin D supplementation, and to evaluate the consistency of the two detection methods, so as to provide references for clinicians.

This study was based on the practicability of vitamin D supplementation strategies in healthy people. Therefore, daily, 10-day, and monthly vitamin D supplementation groups were set, and the selection of monitoring time points was based on the comprehensive consideration of pharmacokinetics and practicability of real-world studies.

## 2 Materials and methods

### 2.1 Research participants

The study participants were 42 healthy adults recruited from the Second Hospital of Hebei Medical University in October 2022. The study subjects were 42 healthy adults recruited by our advertisement in the Second Hospital of Hebei Medical University in October 2022.The inclusion criteria were as follows: (1) healthy adults, (2) age >18 years old, and (3) no special vitamin D supplementation has been given in the past 6 months from the first visit. The exclusion criteria are as follows: (1) presence of congenital or hereditary diseases (phenylketonuria, nodular sclerosis, neurofibromatosis, etc.); (2) significant history of traumatic brain injury and other neurological functional diseases; (3) severe visual and hearing impairment; (4) heart, lung, liver, kidney, endocrine, or autoimmune diseases; (5) gastrointestinal diseases, difficulty eating, and malnutrition; (6) acute or chronic infectious diseases in recent 3 months; and (7) intake of nutritional supplements such as vitamins and minerals in the past 6 months or taking medication recently. For each participant, detailed clinical examinations were conducted, and personal medical history records, including height, weight, body mass index, etc., were collected.

This study was approved by the Ethics Committee of the Second Hospital of Hebei Medical University (Ethical batch no. 2022-R766), the participants signed the informed consent form, and the original study was registered with the China Clinical Trial Registration Center (Registration no. ChiCTR230069387).

### 2.2 Research methods

#### 2.2.1 25(OH)D serum sample collection

Serum samples were collected and sent to the subjects on day 1, day 14, day 28, month 6, and month 12 after the formal start of the study. On the day of sampling, all subjects were in a fasting state, and there was no inflammation or edema in the blood collection area. Venous blood collection and capillary blood collection were carried out successively by the same group of medical personnel. After routine disinfection, disposable venous blood collection needle (sphenwing type, Shandong Zhu Pharmaceutical) was used to puncture the left elbow vein of the subject, and vacuum negative pressure collection vessel was connected (special coagulant and special separation glue were added, Shanghai Aoxiang Medical), 2 ml venous blood was collected and injected into the collection vessel, and centrifuged for 1 h (4,000 r/min, 10 min) and stored away from light. Select the inner tip of the middle or ring finger for capillary blood collection, massage the blood collection site, after routine disinfection, use a disposable blood collection needle (Shandong Zhushe Pharmaceutical) to Pierce the skin 2 mm, wipe off the first drop of blood, use a disposable micro-blood collection straw (Changsha Global Medical Equipment Co., LTD.) to collect 100 μl capillary blood. The blood is squeezed from a microsampling pipette into an EP tube (Eppendorf) containing the anticoagulant EDTA, immediately mixed at least 10 times, and stored at room temperature away from light. All samples will be labeled and stored for future reference, and tested at low temperature on the same day.

From 1 day, 14 days, 28 days, and 6 months after recruitment, two samples of venous serum and two samples of capillary blood were collected on an empty stomach for testing. Venous blood is collected from the cubital vein or jugular vein, with a volume of 2 ml, and injected into a vacuum test tube. Select the inner side of the middle or ring finger tip for capillary blood collection, and collect a blood volume of 100 μl using a disposable micro blood collection pipette. The blood sample was stored at room temperature and away from light. After 1 h, it was centrifuged (4,000 r/min, 10 min) and kept for collection. It was stored at 4°C before being sent for testing. Low-temperature transportation testing was contacted on the same day. Then, 100 μL of capillary blood was collected using a disposable micro blood collection pipette. The blood was squeezed from the micro blood collection vessel into the EP (Eppendorf) tube containing anticoagulant ethylenediaminetetraacetic acid and immediately mixed at least 10 times. The sample was marked and kept for future reference, and low-temperature transportation testing was contacted on the same day.

#### 2.2.2 25(OH)D detection

To reduce the effect of testing instruments and methods on data accuracy, venous serum samples were sent to the laboratory of Beijing Jinyu Testing Center for unified testing. Liquid chromatography-tandem mass spectrometry (LC-MS/MS, 4500MD, AB SCIEX, USA) was used for the detection of serum 25(OH)D levels. Serum 25(OH)D levels in venous and capillary blood samples were measured by chemiluminescence immunoassay using automatic chemiluminescence analyzer (MCL60, Nanjing Renpulse Biotechnology Co., Ltd.). LC-MS/MS is defined as the most recognized “gold standard” for the measurement of 25(OH)D. Internal quality control (IQC) is carried out on all test samples by the tester to ensure that the laboratory test results can be subsequent statistical analysis.

#### 2.2.3 25(OH)D standard deviation score calculation

Using the reference range of serum 25(OH)D levels in the *Chinese Journal of Pediatrics* “Practice Guidelines for Clinical Issues Related to Vitamin D Nutrition in Children” to grade vitamin D nutritional status: Based on serum 25(OH)D levels, vitamin D nutritional status is classified as vitamin D deficiency [25(OH)D < 30 nmol/L] and vitamin D insufficiency [25(OH)D 30–50 nmol/L]. There are four levels of vitamin D adequacy [25(OH)D > 50–250 nmol/L] and vitamin D toxicity [25(OH)D > 250 nmol/L] (3). By establishing a mathematical model, generate polynomial equations for the mean and standard deviation, with SDS = (measured value average)/s.

### 2.3 Statistical methods

Statistical analysis was conducted using IBM SPSS Statistics version 27.0 (IBM Corp., Armonk, NY, USA). The Kolmogorov-Smirnov test (KS test) was used to test the normality of the three groups of data. Pearson correlation analysis was used to analyze the correlation between the results of the two detection methods and the results of the two samples. Pearson correlation analysis and linear regression analysis were used to detect the correlation and determine the correction algorithm. Bland-Altman plot and Kappa statistic were used to analyze the consistency. The concepts of approximate entropy and least squares were added to the curve fitting. The weighted Kappa coefficient of 25(OH)D classification was calculated to analyze the consistency of the three groups of data. Using receiver operating characteristic (ROC) curve method to evaluate the sensitivity and specificity. The results were analyzed according to the *p* value and kappa value, and the difference was statistically significant (two-tailed *P* < 0.05). We use Cronbach α The coefficient (a commonly used reliability analysis method) is used to compare the consistency or stability of the results obtained from the 25(OH)D test of venous blood serum samples and capillary blood serum samples. If the reliability coefficient is above 0.8, the reliability of the experiment or scale is very good; A reliability coefficient above 0.6 is acceptable; If it is below 0.6, the scale needs to be redesigned.

## 3 Results

### 3.1 Basic information

**Figure 2:**
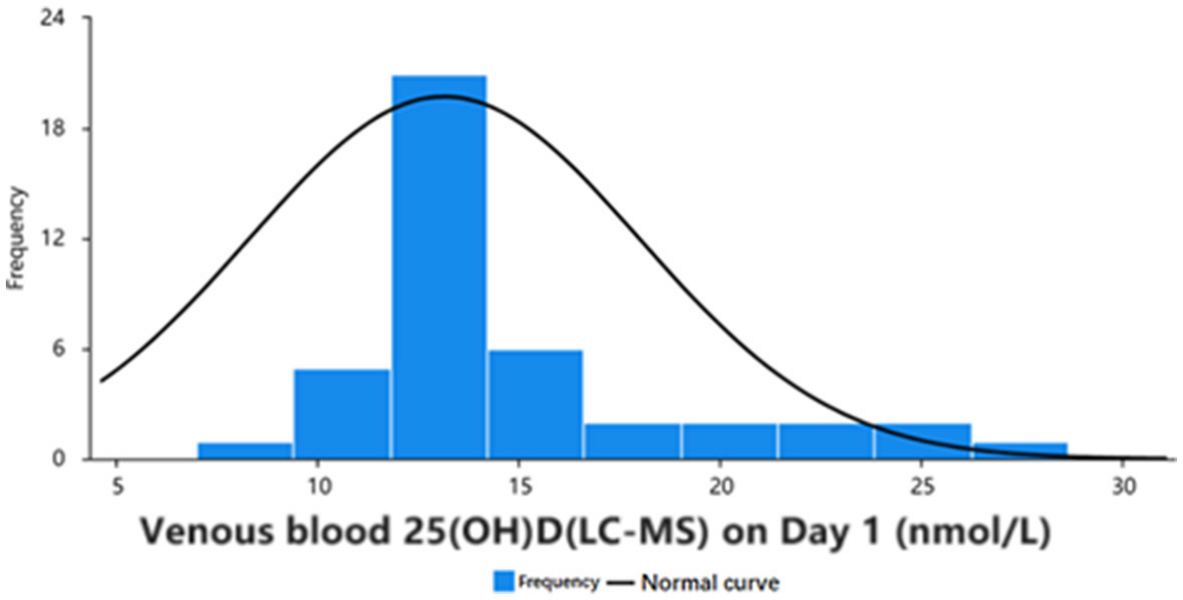
Frequency distribution and normality curve of initial venous blood 25(OH)D concentration in 42 participants.

The serum samples of 42 participants [including 14 men (33%) and 28 women (65%)], including 168 venous blood serum samples and 168 capillary blood serum samples, were statistically compared using 168 pairs. In the 42 samples from day 1 of the study, according to the “Practice Guidelines for Clinical Issues Related to Vitamin D Nutrition in Chinese Children,” 27 participants had vitamin D deficiency, 10 had vitamin D insufficiency, and 5 had vitamin D adequacy (3). Data were analyzed for normality using the Jarque Bra test, and the results showed a normal distribution of 25(OH)D values in the venous blood (Table 1, Figure 2). The mean values of the three groups were: 30.00, 29.93, 29.33 nmol/L, there was no statistical significance (*P* > 0.05). The three groups all reached a stable peak at 6 months, and the average levels of the three groups were 49.21, 42.50, and 43.025 nmol/L, respectively. The average levels of group A were higher than those of group B and group C, and the difference was statistically significant (*P* < 0.001, Table 2).

**Table 1:** Basal metabolic data and body composition analysis data of the subjects.

	**Group A**			**Group B**			**Group C**				
	**Baseline**	**12 m**	***P*** **-value** ^*^	**Baseline**	**12 m**	***P*** **-value** ^*^	**Baseline**	**12 m**	***P*** **-value** ^*^	***P*** **-value** ^**^	**Cohen's d**
Height, cm	168.00 ± 7.63	168.00 ± 7.62	0.854	167.56 ± 3.25	167.55 ± 3.12	0.873	168.21 ± 5.42	168.20 ± 5.41	0.862	0.234	0.00
BMI, kg/m^2^	25.35 ± 0.18	25.30 ± 0.22	0.01	22.46 ± 0.35	22.44 ± 0.46	0.04	21.35 ± 0.26	21.30 ± 0.11	0.001	0.001	0.10
Body fat, kg	15.14 ± 3.38	16.22 ± 4.24	< 0.001	18.46 ± 2.54	18.57 ± 2.44	0.03	19.33 ± 3.24	17.24 ± 3.22	< 0.01	0.01	0.14
Body fat%	33.74 ± 10.25	36.58 ± 0.54	< 0.001	34.33 ± 4.24	34.45 ± 5.66	0.01	31.25 ± 11.22	29.51 ± 9.0.35	< 0.01	0.001	0.01
Total body water, kg	34.46 ± 5.62	34.50 ± 4.82	0.24	34.25 ± 3.65	34.17 ± 2.51	0.38	34.68 ± 2.65	34.70 ± 2.51	0.28	0.09	0.07
Waist-to-hip ratio	0.87 ± 0.18	0.88 ± 0.17	0.45	0.85 ± 0.25	0.85 ± 0.65	0.77	0.83 ± 0.31	0.82 ± 0.27	0.64	0.15	0.06
Bone mass, kg	3.07 ± 0.49	3.44 ± 0.58	< 0.01	3.24 ± 0.22	3.36 ± 0.68	< 0.01	3.80 ± 0.25	3.85 ± 0.22	< 0.01	< 0.01	0.16

^*^p < 0.05, ^**^p < 0.01.

**Table 2:** Summaries of statistical results from each time period (nmol/L).

	**Group A**	**Group B**	**Group C**	**Total**
Day 1	30.00 ± 3.472	29.93 ± 3.64	29.33 ± 2.88	29.912 ± 3.468
Day 14	38.52 ± 3.63	39.54 ± 3.26	39.66 ± 2.90	39.712 ± 3.208
Day 28	41.32 ± 2.87	40.74 ± 2.866	40.98 ± 2.45	40.886 ± 3.104
Month 6	49.21 ± 2.74	42.50 ± 3.29	43.03 ± 2.03	44.943 ± 2.930
Month 12	48.75 ± 3.58	42.88 ± 4.11	43.22 ± 1.58	45.136 ± 3.393

The levels of vitamin D in venous blood with two different detection methods and capillary blood with chemiluminescence method were analyzed by paired *t*-test. The paired *t-*test showed that the vitamin D levels in venous blood detected by mass spectrometry and capillary blood detected by chemiluminescence method were significantly different (*t* = 8.326, *p* < 0.01) (Table 3). Paired *t*-test showed that the difference of 25(OH)D levels in venous blood and capillary blood detected by chemiluminescence method was also statistically significant (*t* = 5.636, *P* < 0.05), and the mean value of capillary 25(OH)D level was significantly higher than that of venous 25(OH)D level detected by the two methods (*P* < 0.05).

**Table 3:** Paired *T*-test analysis results for venous blood and capillary blood 25(OH)D concentration.

**Item**	**Mean value**	**Standard deviation**	**t**	**P**
Venous blood 25(OH)D LC-MS nmol/L	45.32	8.56		
			8.326	< 0.01
Capillary blood 25(OH)D LC-MS CLIA nmol/L	52.03	7.44		
			5.636	< 0.05
Venous blood 25(OH)D LC-MS nmol/L	49.88	12.66		

25(OH)D concentration.

### 3.2 Consistency analysis of the two detection samples

**Figure 3:**
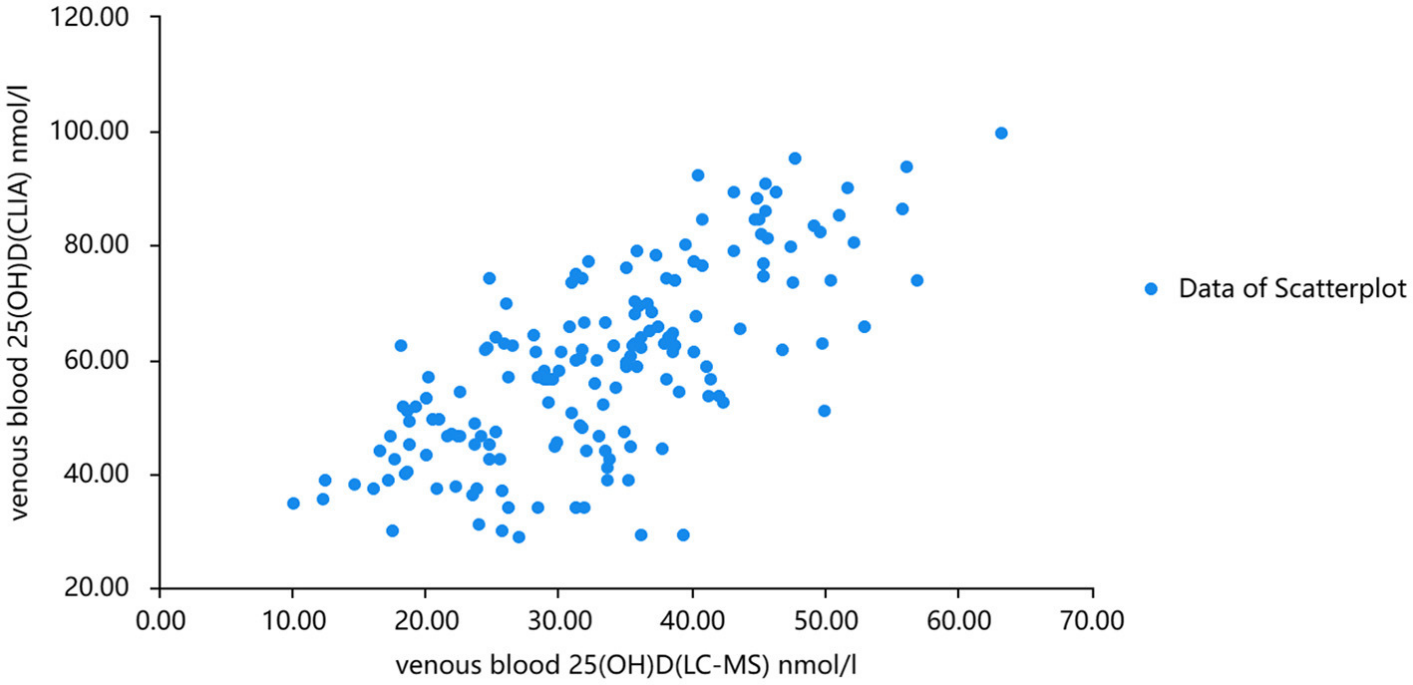
Scatter plots of serum 25(OH)D levels analyzed by mass spectrometry and chemiluminescence, respectively.

**Figure 4:**
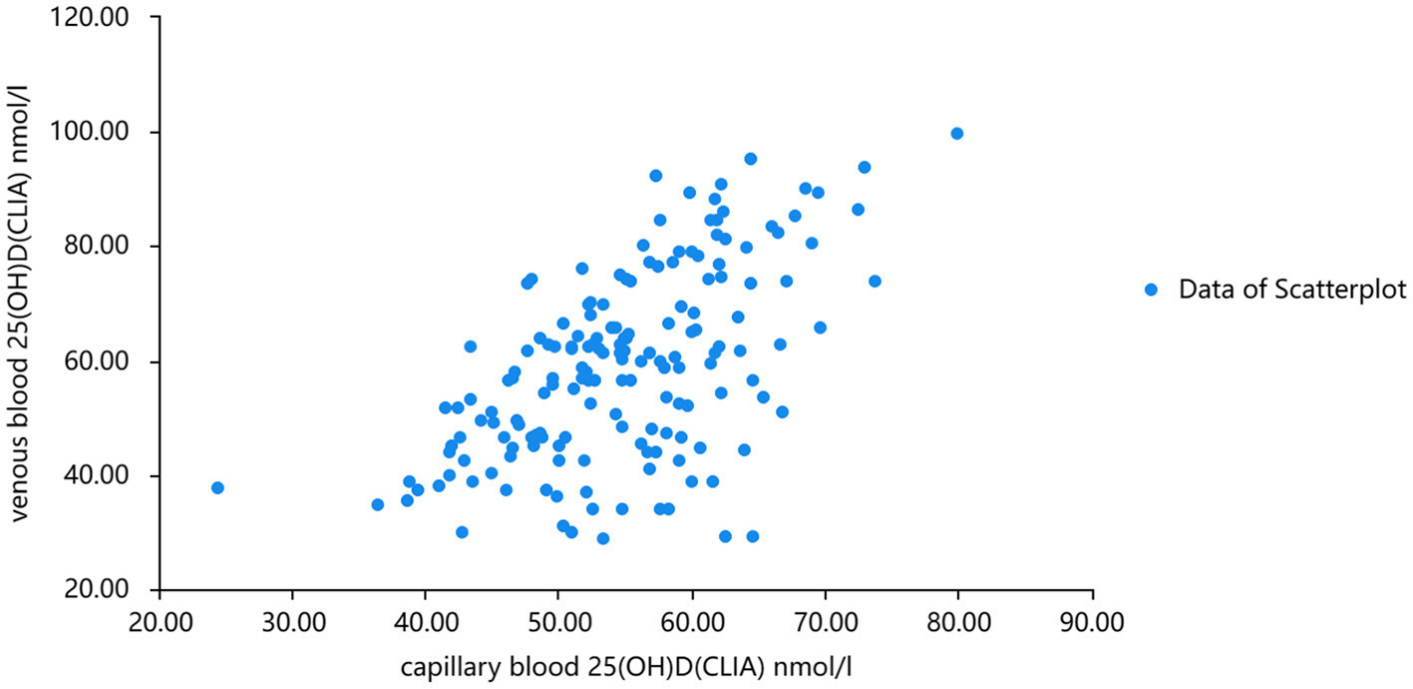
Chemiluminescence was used to analyze the scatter plots of venous 2 and capillary 25(OH)D concentrations.

The linear fitting formula of scatter data from venous blood detected by chemiluminescence and mass spectrometry was as follows: venous blood 25(OH)D(CLIA) nmol/l =1.128^*^venous blood 25(OH)D(LC-MS) + 3.628 nmol/l R^2^ = 0.702. Normality test was carried out on the three groups of data, and all of them were normal distribution. Pearson correlation analysis showed that there was a good correlation between 25(OH)D levels measured by the two methods (r = 0.732, *P* < 0.001). When the concentration of 25(OH)D in venous and capillary samples was measured by chemiluminescence, the concentration of 25(OH)D in capillary blood was higher than that in venous (Figures 3, 4). The linear fitting formula of scatter data measured by chemiluminescence was as follows: concentration of 25(OH)D in venous blood (nmol/L) = 1.105 ^*^ concentration of 25(OH)D in capillary blood −7.532 nmol/L, R^2^ = 0.625.

**Figure 5:**
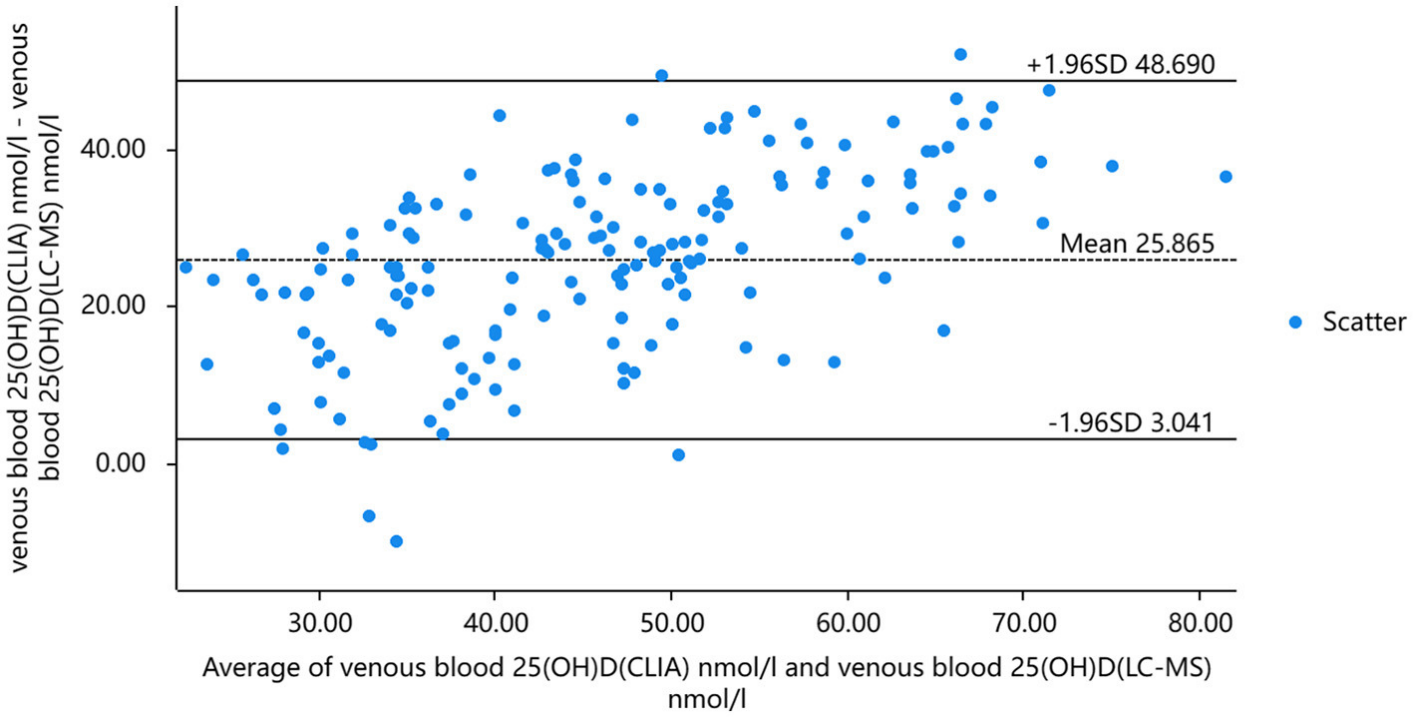
The results of 25(OH)D in venous blood were analyzed by mass spectrometry and chemiluminescence and Bland-Altman analysis.

**Figure 6:**
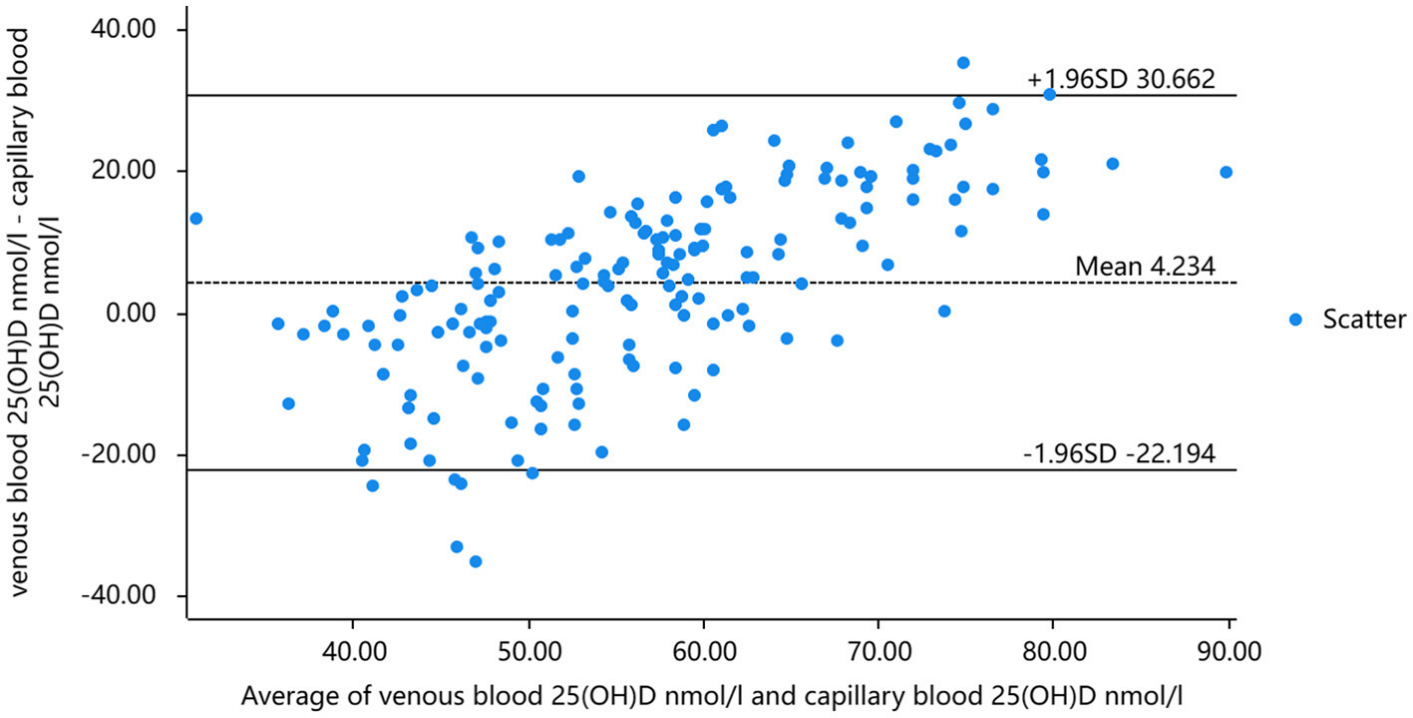
Bland-Altman analysis of 25(OH)D concentration in venous and capillary blood by chemiluminescence method.

Taking into account that the variability of differences increases with amplitude, we plotted the percentage difference on the Bland-Altman. Analysis revealed a significant positive bias in venous blood 25(OH)D measured by CLIA compared to LC-MS. The results of CLIA were significantly higher than those of LC-MS. The standard deviation of the difference was 4.259 nmol/ml (95% confidence interval [CI] −2.013 to 8.465), which translates to a percentage difference of 6.259%. Bland-Altman analysis showed that the serum 25(OH)D of capillary blood and venous blood measured by CLIA method had a significant positive bias, and the detection result of capillary blood was significantly higher than that of venous blood. The standard deviation of the difference was 2.234 nmol/mL (95% confidence interval [CI] −10.662–15.130), which translates to a percentage difference of 1.266% (Figures 5, 6).

In Cronbach's reliability analysis, the reliability coefficient value was 0.765 which was >0.6, indicating that the reliability quality of the research data was acceptable. For the “CITC value,” the values of the analysis items were all >0.4, indicating a good correlation between the analysis items and a good level of reliability. In summary, the reliability coefficient value of the research data was >0.6, indicating that the quality of data reliability was acceptable.

We used approximate entropy, a non-linear dynamic complexity measure, to evaluate the efficacy of vitamin D treatment. The approximate entropy of other variables to vitamin D ratio was measured, with a *T*-value of 0.4614 for bone density, 0.5864 for vitamin A, and 0.7462 for capillary blood vitamin D. Among other possibly related analysis results, the correlation between vitamin D in the capillary blood and venous blood was good.

Kappa statistic analysis was used to evaluate the consistency of the results of vitamin D detection among the above three groups of data. When the data were classified as vitamin D sufficient, insufficient, and deficient, paired analysis was performed for the comparison between the three groups of data (Table 4). The *p* values of the three groups of data were all < 0.01, and the Kappa value was >0.7, indicating that the results of 25(OH)D analysis in venous blood by chemiluminescence method were consistent with the results of 25(OH)D analysis in capillary blood by mass spectrometry. Combined with the Kappa coefficient analysis, the Kappa coefficient of the results of venous blood 25(OH)D analysis by chemiluminescence method and capillary blood 25(OH)D analysis by mass spectrometry was 0.723, which was between 0.6 and 0.8, indicating that the results of the two groups had a strong consistency in judging whether vitamin D deficiency and the results were reliable.

**Table 4:** Kappa coefficient table for the consistency evaluation of the three 25(OH)D results.

**Item**	**Kappa value**	**Standard error**	***Z* value**	*p* value^*^	**95%CI**
Venous blood 25(OH)D(LC-MS) & capillary blood 25(OH)D(CLIA)	0.723	0.066	4.890	0.01	0.203–0.443
Venous blood 25(OH)D(LC-MS) & venous blood 25(OH)D(CLIA)	0.895	0.071	8.431	0.01	0485–0.704
Venous blood 25(OH)D(LC-MS) & capillary blood 25(OH)D(LC-MS)	0.793	0.063	6.730	0.01	0.403–0.653

The 95% confidence interval calculation formula in Table 4 has been added to the note of the table. 95%CI = [Kappa value ± 1.96^*^ (progressive standardized error)]. ^*^p < 0.05.

**Figure 7:**
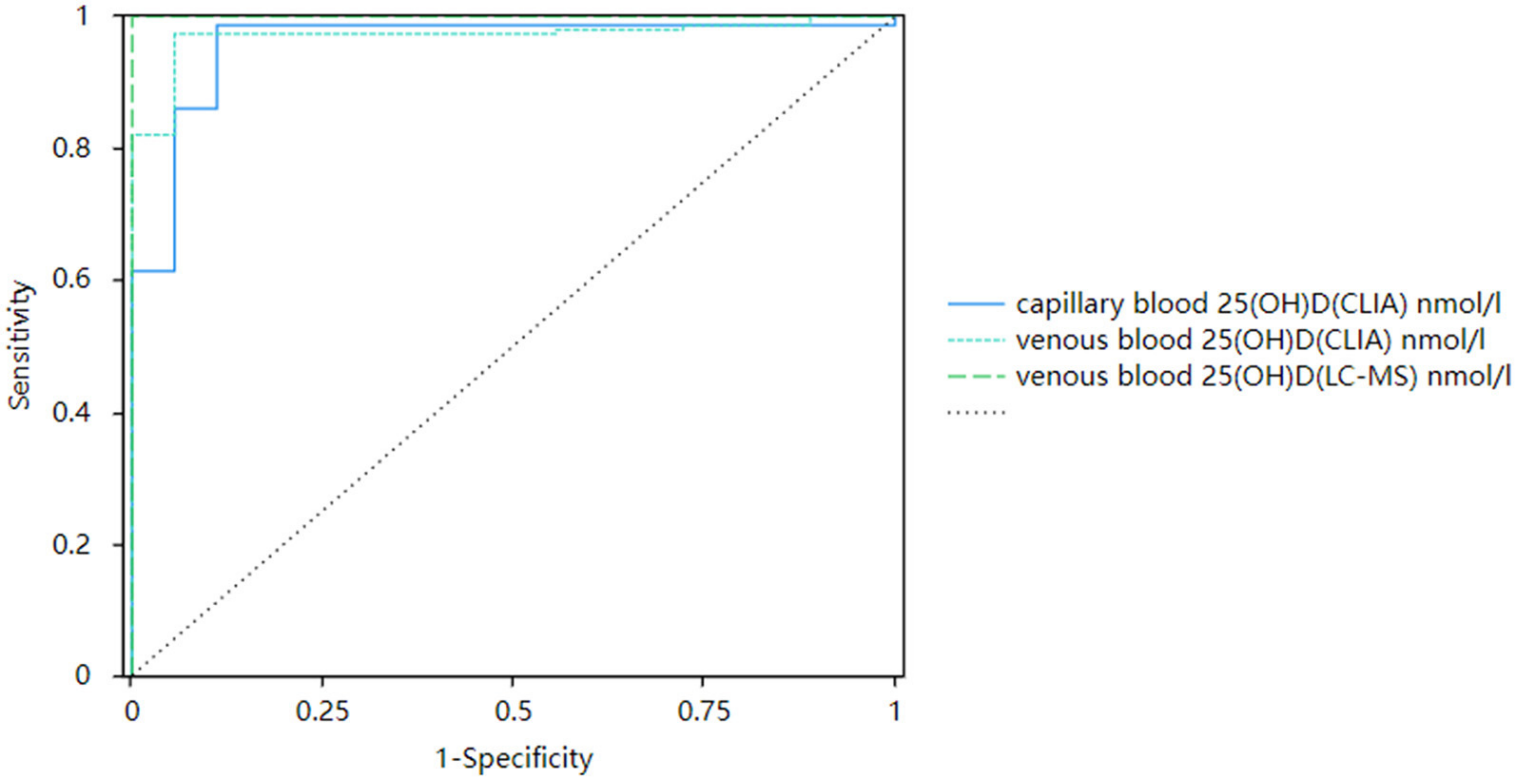
Corrected receiver operating curves of capillary and venous 25(OH)D with 30 nmol/L as the cutoff value.

**Figure 8:**
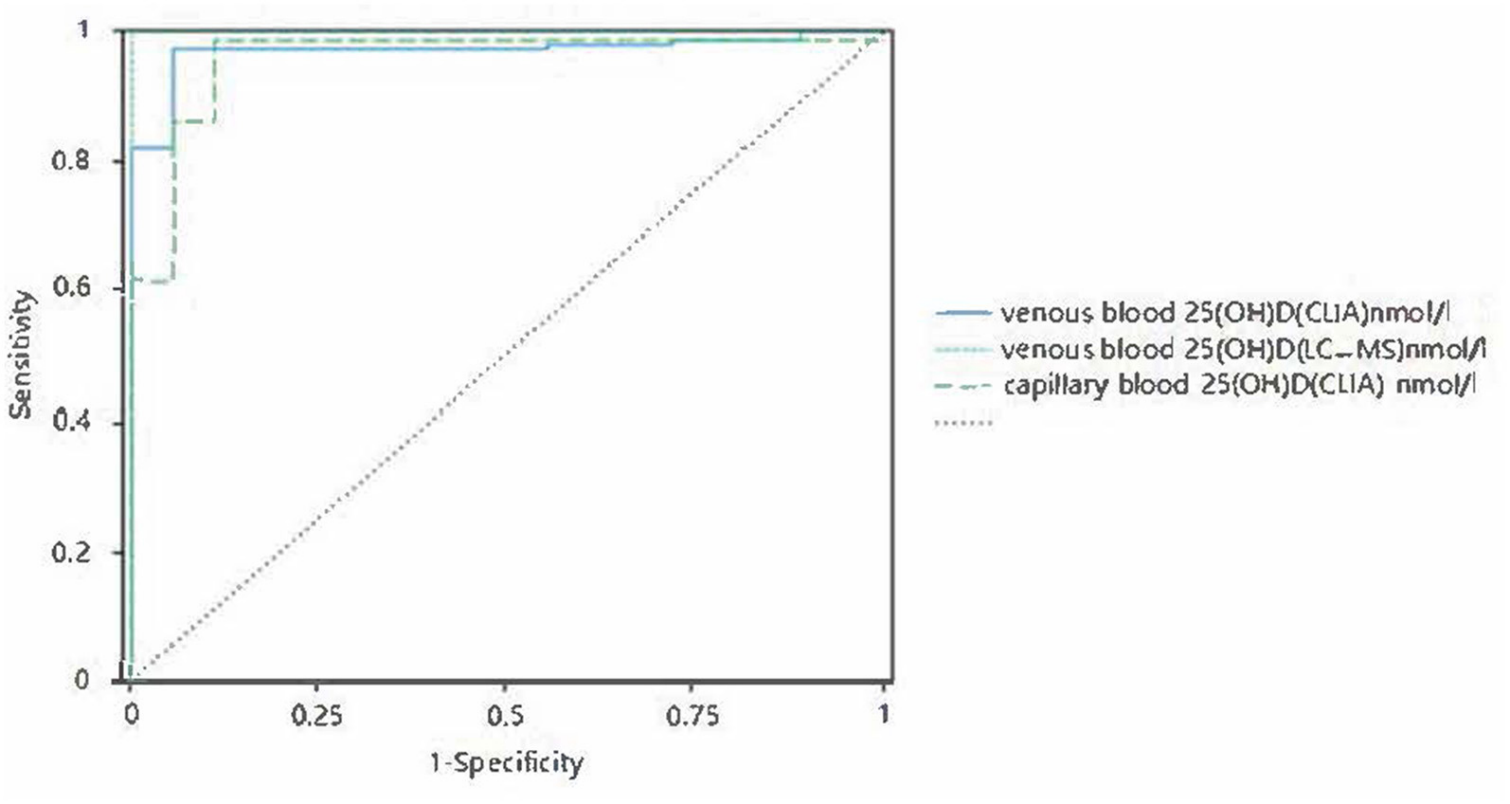
Corrected receiver operating curves of capillary and venous 25(OH)D with 50 nmol/L as the cutoff value.

There was a significant positive correlation between capillary blood serum 25(OH)D and venous blood serum 25(OH)D. When the corrected capillary serum 25(OH)D was used to diagnose vitamin D deficiency, the capillary serum 25(OH)D result was able to identify subjects with vitamin D deficiency (clinical thresholds 30.00 and 50.00 nmol/L) with sensitivity of 0.97–0.98,. The specificity was 0.89–0.94, and the ROC AUC was 0.96–0.97 (*P* < 0.001, Figures 7, 8).

## 4 Discussion

Vitamin D is an important nutrient that maintains bone metabolism in the human body. Vitamin D deficiency is closely related to chronic diseases such as multiple sclerosis, type I and II diabetes (6). Conversely, vitamin D deficiency increases the risk of preeclampsia, and lower serum 25(OH)D concentrations are associated with cardiovascular diseases in pregnant women and their offspring (7). 25(OH)D is an active form of vitamin D produced by liver hydroxylation in the body. It is relatively stable in the blood and has no significant pulse secretion or circadian rhythm changes. It is the best indicator for evaluating the vitamin D nutritional status in the body (8). Many studies on this topic have been conducted in different directions both domestically and internationally (9–11). The accurate detection of 25(OH)D and the correct interpretation by clinical doctors are of great significance to monitor the efficacy and safety of vitamin D deficiency-related diseases in the diagnosis and treatment process.

There are consistent assessments of serum 25(OH)D levels in venous and capillary blood, and consistent assessments of 25(OH)D levels in the same blood sample by different assays. A study assessing vitamin D nutritional status in newborns indicated that two common immunoassays lead to very different classifications of vitamin D status. May be related to interference with other vitamin D metabolites (12). In other studies, although there was good agreement between capillary blood and venous blood measurements, the subjects were mostly children, and capillary blood 25(OH)D measurements were lower than venous blood measurements (13).

The detection methods for serum 25(OH)D range from initial radioimmunoassay and enzyme-linked immunosorbent assays to high-performance liquid chromatography, electrochemiluminescence immunoassay (ECLISA), and LC-MS/MS, which have become increasingly accurate methods for detecting serum 25(OH)D levels. At present, the mainstream detection methods are ECLISA and LC-MS/MS. LC-MS has higher specificity in detecting 25(OH)D, which can more accurately distinguish 25(OH)D from other similar compounds, improving the sensitivity and accuracy of detection. This reduces the possibility of cross-reactivity and non-specific binding, and the measurement results are generally considered to be closer to the actual level. CLIA uses antibodies, and other vitamin D metabolites with structures similar to 25(OH)D are also likely to cross-react, leading to higher CLIA measurements. Therefore, there may be significant differences in the results of 25(OH)D determination between the two methods.

Some clinicians use immunoassay to detect 25(OH)D. If the serum 25(OH)D standard used in the determination of vitamin D nutritional level is consistent with CLIA method, the vitamin D status of patients may be misjudged. Therefore, caution must be exercised when interpreting the serum 25(OH)D detection report and the detection method must be clearly defined. In this trial, we tried to establish a linear relationship between the different results in order to obtain a more accurate vitamin D level.

According to the test results, the result of serum 25(OH)D in venous blood measured by CLIA method was greater than that of capillary blood serum 25(OH)D measured by CLIA method. The clinician may not be able to determine the vitamin D level of the patient due to different methods or different samples using the same judgment criteria, so we suggest using the truncation value derived from the linear equation for vitamin D status assessment: The cut-off values of 25(OH)D in venous blood were 30.00, 50.00, and 250.00 nmol/L by LC-MS, and the corresponding cut-off values of 25(OH)D in venous blood serum by CLIA method were 37.468, 60.028, and 285.628 nmol/L, respectively. The cut-off values of 25(OH)D in capillary blood by CLIA were 40.723, 66.844, and 265.303 nmol/L, respectively.

When detecting vitamin D, venous blood volume of 3–5 mL must be collected, and the capillary blood volume must be 20 μL. The amount of capillary blood collected is small (13), the tools used are simple, the detection process is time-consuming, and it is more convenient when the patient cannot, or is not suitable, or have conditions that do not support it. Clinicians are faced with different blood collection methods, and their concentration relationships must be compared. In this study, the two detection methods showed good consistency when the measured value of 25(OH)D was >37.5 nmol/L, and the two can be mutually referenced. However, for capillary blood, during the collection process, owing to the possibility of infiltration of the squeezed fabric into the collected samples, different vitamin D levels in different body parts, and the susceptibility of capillary blood examination results to environmental temperature, humidity, and other factors may lead to certain deviations in the test results, and the results cannot be distinguished between D2 and D3, which must be taken into consideration.

## 5 Conclusions

In summary, the detection results of 25(OH)D in the capillary and venous blood are comparable. When venous blood collection is inconvenient or there are special requirements for blood collection volume and frequency, the vitamin D level in capillary blood samples can be measured to replace the venous blood. The obtained results can be calculated using a common formula to determine the vitamin D content in the venous blood or converted into a reference range for the capillary blood. However, owing to the small sample size of this study, the normal distribution of 25(OH)D in the serum of the participants did not show extreme values. The detection method, data conversion, and result interoperability must be confirmed, and the feasibility still needs to be further expanded to provide more accurate references for clinical practice.

## Data availability statement

The original contributions presented in the study are included in the article/supplementary material, further inquiries can be directed to the corresponding author.

## Ethics statement

The studies involving humans were approved by the Ethics Committee of the Second Hospital of Hebei Medical University. The studies were conducted in accordance with the local legislation and institutional requirements. The participants provided their written informed consent to participate in this study.

## Author contributions

YX: Conceptualization, Formal analysis, Methodology, Writing – original draft, Writing – review & editing. KW: Writing – original draft, Investigation, Software. XM: Writing – review & editing, Validation. HZ: Conceptualization, Visualization, Writing – review & editing. XT: Data curation, Project administration, Writing – review & editing.
